# Chlorzoxazone, a small molecule drug, augments immunosuppressive capacity of mesenchymal stem cells via modulation of FOXO3 phosphorylation

**DOI:** 10.1038/s41419-020-2357-8

**Published:** 2020-03-02

**Authors:** Luchan Deng, Hongling Li, Xiaodong Su, Yingjie Zhang, Haoying Xu, Linyuan Fan, Junfen Fan, Qin Han, Xueyuan Bai, Robert Chunhua Zhao

**Affiliations:** 1 Center of Excellence in Tissue Engineering, Institute of Basic Medical Sciences and School of Basic Medicine, Chinese Academy of Medical Sciences and Peking Union Medical College, Beijing Key Laboratory of New Drug Development and Clinical Trial of Stem Cell Therapy (BZ0381), Beijing, 100005 PR China; 2 Brain Tumor Research Center, Beijing Neurosurgical Institute, Beijing Tiantan Hospital Affliated to Capital Medical University, Beijing Laboratory of Biomedical Materials, Beijing, 100007 PR China; 3 Department of Nephrology, Chinese PLA General Hospital, Chinese PLA Institute of Nephrology, State Key Laboratory of Kidney Diseases, National Clinical Research Center for Kidney Diseases, Beijing Key Laboratory of Kidney Diseases, Beijing, 100853 PR China; 4 Institute of Basic Medical Sciences Chinese Academy of Medical Sciences, School of Basic Medicine Peking Union Medical College, Peking Union Medical College Hospital, Beijing Key Laboratory of New Drug Development and Clinical Trial of Stem Cell Therapy (BZ0381); Qingdao University, Qingdao, PR China

**Keywords:** Phosphoinositol signalling, Mesenchymal stem cells

## Abstract

Nowadays, immune diseases are a large burden in healthcare. Mesenchymal stem cells (MSCs) have prominent ability in immunomodulation and have been applicated on treating many immune-related diseases. However, the clinical outcomes can be disparate and sometimes completely counterproductive beyond explanation of cell heterogeneity. The theory of immunomodulation plasticity in MSCs has then emerged to explain that MSCs can be induced into proinflammatory MSC1 or anti-inflammatory MSC2 responding to different immune environment. It would be safer and more efficient if we could induce MSCs into a certain immune phenotype, in most cases MSC2, prior to medical treatment. In this study, we screened and identified a classical FDA-approved drug, chlorzoxazone (CZ). Unlike traditional method induced by IFN-γ, CZ can induce MSC into MSC2 phenotype and enhance the immunosuppressive capacity without elevation of immunogenicity of MSCs. CZ-treated MSCs can better inhibit T cells activation and proliferation, promote expression of IDO and other immune mediators in vitro, and alleviate inflammatory infiltration and tissue damage in acute kidney injury rat model more effectively. Moreover, we discovered that CZ modulates phosphorylation of transcriptional factor forkhead box O3 (FOXO3) independent of classical AKT or ERK signaling pathways, to promote expression of downstream immune-related genes, therefore contributing to augmentation of MSCs immunosuppressive capacity. Our study established a novel and effective approach to induce MSC2, which is ready for clinical application.

## Introduction

Immune diseases have become a large burden of population worldwide. So far, there is no effective cure for most immune diseases other than some remedies as symptomatic and supportive treatment^1^. Acute tissue injuries often cause excessive immune response, sometimes lead to organ failure and contribute to cellular senescence even healed^2^. Mesenchymal stem cells (MSCs) have been proved to be highly effective in improving health condition and prognosis of patients suffering from immune-related diseases and acute tissue injuries, such as graft-versus-host disease^3^, autoimmune diseases^4–6^, kidney, liver, and heart injury^7–9^, due to their prominent ability in immune regulation, mainly immune suppression by inhibiting proliferation and function of almost all types of immune cells except for regulatory T cells which are generally supported by MSCs^10,11^.

However, despite potent effectiveness of MSCs in immune regulation, problems are gradually emerging as MSCs widely been applied in medical field. Gallipeau’s team observed that MSCs from different donors showed conspicuous discrepancies in immune suppressive activities during clinical trials^12^. Another clinical trial conducted by Teerlink et al. showed that some patients with congestive heart failure got worse outcome when receiving higher dose of autologous MSCs therapy^13^. An animal study from our lab suggested that MSCs can aggregate collagen-induced rheumatic arthritis^14^. And oddly enough, some researchers did prove MSCs to suppress tumor progression and migration^15–17^, while others found that MSCs support cancer growth and act as an accomplice through tumor metastasis and deterioration^18–20^. These contradictory results are beyond simple explanation of heterogeneity, bringing deep concern about the medical application of MSCs. It is urgent to study further about the immunomodulatory mechanism of MSCs in order to rein the mischievous cells to treat diseases effectively and safely.

To unravel these discrepancies, accumulating evidences gleaned in labs worldwide indicated that there are two distinct types of MSCs in human body which can switch their immune functions responding to microenvironment and inflammatory status. Since Waterman et al. first identified these two polarized MSCs as proinflammatory MSC1 and anti-inflammatory MSC2 in 2010^21^, there have been plentiful studies focused on the immune plasticity of MSCs and scientists are zealous in harnessing the unique property of MSCs to optimize medical application^22,23^.

It would be more efficient and safer in clinical application if we could induce MSCs to a certain immune type, in most cases MSC2, prior to infusion to patients. Current induction system for MSC2 includes IFN-γ (together with TNF-α) and polyinosinic acid–polycytidylic acid (poly(I:C)), but they dramatically increase expression of immunogenic markers on MSCs^24^, rendering them not suitable for clinical use. Small molecule drugs are easy to deliver and safe to use on bedside since most of them are long been scrutinized by supervising agencies and worldwide market^25^. Many classical drugs have been found a novel target in recent years, bringing new hope to some refractory diseases. Therefore, we undertake the task to identify a classical drug that may enhance the immune function of MSCs.

Through rigorous and systemic analysis, we were able to screen out a drug, chlorzoxazone (CZ), from dozens of potential candidates in FDA-approved drug library. In this study, we demonstrate that CZ can effectively promote the immunosuppressive ability of MSCs on T-cell activation and proliferation in vitro. Furthermore, CZ-treated MSCs can alleviate inflammatory infiltration in renal tissue and lessen fibrinoid necrosis of glomeruli more effectively in vivo. CZ inhibits the phosphorylation of transcriptional factor forkhead box O3 (FOXO3), potentially resulting in higher expression of anti-inflammation cytokines, chemokines, and enzyme indoleamine 2,3-dioxygenase (IDO), which are essential in MSC-mediated immune suppression process.

## Materials and methods

### Key reagents

Drug library, CZ, kinase inhibitors, and activators were purchased from TargetMol (Boston, MA, USA) and Selleck Chemicals (Houston, TX, USA). IFN-γ and TNF-α were purchased from PeproTech (Rocky Hill, NJ, USA). Poly(I:C) were purchased from Sigma-Aldrich (St Louis, MO, USA).

### Drug screening

A computer analysis platform created from Cui Lab in Peking University, Beijing, China, was introduced to grade and rank drugs from FDA-approved drug library based on their relevance to immune function. Then top 20 and bottom 10 drugs were selected to proceed following functional studies to lock the final expected drug. Screening platform website: http://www.cuilab.cn/drugpattern.

### MSCs preparation

Human MSCs were isolated from human umbilical cord as previously described^26^ under approval of the Ethics Committee of the Chinese Academy of Medical Sciences and Peking Union Medical College and informed consent of each donor. Tissue was collected from full-term human placentas through caesarean section with informed consent. In brief, the umbilical cord was washed thoroughly with phosphate buffer saline (PBS) for several times before cutting into segments of 5 cm, then the segments were longitudinally cut open and all the blood vessels were carefully removed. The segments were cut into small cubes around 1–2 mm^3^ and then 0.4% collagenase P (Roche, Switzerland) was added to digest the tissue while gently shaking for 1 h. Cells were obtained by centrifugation after filtrating through 100 μm filter (BD Bionsciences, San Jose, CA, USA) and washing for two times with PBS.

Cells were cultured on T75 flasks (Thermo-Fisher Scientific, USA) in DMEM/F12 medium (Corning, USA) with 2% FBS (Gibco, Grand Island, NY, USA), 10 ng/ml platelet-derived growth factor bb (PDGF-bb; Sigma-Aldrich), 10 ng/ml epidermal growth factor (EGF; Sigma-Aldrich), 10^–4^ Mascorbic acid 2-phosphate (Sigma-Aldrich), 10^–8^ M dexamethasone (Sigma-Aldrich), 10^–9^ M insulin transferrin selenium (ITS; Gibco), 100 U/ml penicillin, and 100 μg/ml streptomycin (Gibco). T75 flasks were placed in a humidified incubator at 37 °C with 5% CO_2_. Culture medium was replaced every 2–3 days to remove cell debris and only adherent cells were allowed to expand. About 1 week later, cells were harvested for subculture or cryopreservation when cells were more than 90% confluent.

### Peripheral blood monocytes (PBMCs) isolation and co-culture with MSCs

Human PBMCs were isolated as described previously^27^. Transwell plates (0.4 μm pore size, Corning, USA) were used for co-culture system. MSCs were counted and seeded in the lower plates following 10 Gy of radiation to stop DNA duplication and cell proliferation. Then PBMCs were counted and cultured in the upper chambers (MSCs: PBMC = 1:10). The co-culture medium was composed of RPMI1640 (Corning), 10% fetal bovine serum (FBS, Gibco), 1 mM glutamine, 1% nonessential amino acids (NEAA, Sigma-Aldrich), and 5 ng/ml IL-2 (PeproTech). Different reagents or inhibitors/activator were added to the co-culture system when needed.

### T cells activation assay

PBMCs were nonspecifically activated by adding 50 μg/ml polyhydroxyalkanoates (PHA, Roche) in the co-culture system. CD69 and CD25, markers of early-stage and mid-stage activated T cells were detected after 24 and 48 h by flow cytometry. For each experiment, more than three biological repetitive tests were carried out.

### Flow cytometry analysis

Flow cytometric analysis was performed as previously described^28^. In brief, T cells were harvested from transwell chambers and washed twice before incubating with fluorescein-conjoncted antibodies or isotype antibodies (BD Biosciences) for 20 min at 4 °C avoiding from light. Cells were then washed twice prior to perform on Accuri C6 flow cytometers (BD Biosciences) and analyzed by CFlow Plus software (BD Biosciences).

### Carboxyfluorescein succinimidyl ester (CFSE) proliferation assay

PBMCs were dyed by CFSE living cells dye (Biolegend, San Diego, CA, USA) for 30 min avoiding from light at 4 °C prior to T cells and MSCs co-culture. After 3 days, T cells were harvested for flow cytometry to examine the change of fluorescence intensity, and then analyzed by ModFit software (Verity Software House, USA). Cells with higher fluorescence were identified as parent generation. For each experiment, more than three biological repetitive tests were carried out.

### MTS proliferation assay

PBMCs were placed in 96-well plates for 1 × 10^4^ each well after MSCs being seeded and radiated at the bottom of the plates for 1000 each well. MTS reagent (Promega, USA) was added to the culture system at different time points in different plates. After 3 h incubation with MTS reagent in humidified, 5% CO_2_ atmosphere, cell absorbance was recorded by 96-well reader at 490 nm wavelength. Proliferation curve was established according to relative MTS values (each MTS value–Ctrl MTS value) from day 0 to day 4 using GraphPad Prism 8 software (GraphPad Software Inc, USA). For drug screening, cells were subjected to cell absorbance detection only on day 2. For each experiment, more than 3 biological repetitive tests were carried out.

### Induction of different subtypes of T helper (Th) cells

For Th0 maintenance, 10 μg/ml anti-IL-4 (Abcam, Cambridge, MA, USA) and 10 μg/ml anti-IFN-γ (R&D system, Minneapolis, MN, USA) antibodies were added to the culture system; for Th1 induction, 10 ng/ml IL-12 (PreproTech), 10 ng/ml IL-2 (PreproTech), and 10 μg/ml anti-IL-4 antibody were added to the culture system; for Th2 induction, 10 ng/ml IL-4 (PreproTech), 5 ng/ml IL-2, 10 μg/ml anti-IL-12 (Biolegend), and 10 μg/ml anti-IFN-γ were added to the culture system; for Treg induction, 5 ng/ml IL-2 and 5 ng/ml TGF-β (PreproTech) were added to the culture system. T cells were all specifically activated by CD3/CD28 antibodies (BD biosciences).

### RNA isolation and quantitative RT-PCR

Total RNA was extracted from cultured MSCs with TRIzol reagent (Invitrogen, USA) according to manufacturer’s instructions and then quantified by spectrophotometric quantification with NanoDrop 2000 (Thermo-Fisher Scientific, USA). RNA was reverse transcribed by the Reverse Transcription kit (Takara, Japan) according to the manufacturer’s instructions. Quantitative RT-PCR was performed with SYBR premix Ex Taq (Takara) using QuantStudio 3 (Applied Biosystems) in a 10 μl reaction volume. Data were analyzed with QuantStudio Design and analysis software (Applied Biosystems, USA). Samples were run in triplicate. GAPDH was used as an endogenous control. The primers used in this study are listed in Supplementary Table 1. For each experiment, more than three biological repetitive tests were carried out.

### Protein extraction and western blotting

Cells were washed twice with cold PBS. Total protein was extracted by RIPA lysis buffer accompanied with 1 mM PMSF proteinase inhibitors cocktail (Beyotime, China) and phosphatase inhibitors cocktail (Yeasen, China) and then quantified using a BCA Protein Assay kit (Beyotime). Western blotting was performed as previously described^29^. Protein extracts (20 μg) were separated by 10% SDS-PAGE and then transferred to 0.45 µm polyvinylidene fluoride (PVDF) membranes (Millipore). After blocking in 5% bovine serum albumin (BSA) for 1 h at room temperature, the membranes were incubated with specific primary antibodies (FOXO3: ProteinTech, 10849-1-AP; phosphor-FOXO3 (p-FOXO3): Cell Signaling Technology (CST), 5538; ERK: CST, 4695; phosphor-ERK (p-ERK): CST, 4377; AKT: CST, 4691; phosphor-AKT (p-AKT): CST, 9277; GAPDH: ProteinTech, 10494-1-AP) at 4 °C overnight. Then the membranes were incubated with horseradish peroxidase-conjugated secondary antibodies (Neobioscience, China) for 1 h at room temperature. Protein bands were visualized by chemiluminescent ECL reagent (Millipore, Danvers, MA, USA) using Tanon 4600 Chemiluminescent Imaging System (Tanon, China). For each experiment, more than three biological repetitive tests were carried out.

### Immunofluorescence

Cells were fixed by 4% formaldehyde for 15 min then permeabilized by 0.2% Triton X-100 (Sigma-Aldrich) for 3 min. Five percent BSA in PBS was then added to block for 1 h at room temperature. The cells were then incubated with anti-FOXO3 antibody (1:100, Proteintech, 10849-1-AP) overnight at 4 °C. The next day, after gently washing 3 times with PBS for 5 min each, the cells were incubated with secondary antibody (1:500, Invitrogen, USA, A32731) for 1 h at room temperature avoiding from light. After washing with PBS, the chamber with cells were dried and mounted with DAPI (Invitrogen). Images were recorded by an inverted fluorescence microscope (Olympus, Japan).

### Animal experiments

All animal experiments were performed with approval of Institutional Animal Care and Use Committee of the Chinese PLA General Hospital. All Wistar rats were purchased from SPF Biotechnology Co., Ltd (Beijing, China). Rats were housed under SPF conditions for at least 48 h prior to any experiment in the laboratory animal center of the Chinese PLA General Hospital (Beijing, China). Experiments used male Wistar rats of 7–8 weeks, weight range from 215 to 246 g. Six rats were randomly allocated to each group. Thy1.1 antibody (Sigma-Aldrich) was used to induce acute nephritis at 2.5 mg/kg for 2 days prior to MSCs infusion. After 3 days of induction, blood and urine sample were collected before rats were terminated and then kidneys were gathered for pathological section followed by periodic acid–schiff (PAS) staining. Investigators were not blinded to the group allocation.

### Statistical analysis

Data were analyzed by GraphPad Prism 8 software (GraphPad Software Inc. USA). Student’s *t* tests (two-sided) and one-way ANOVA were employed to analyze between two groups or among multiple groups, respectively. *P* < 0.05 was considered statistically significant otherwise not significant. Asterisks were used to indicate as follows: *P < 0.05, **P < 0.01, ***P < 0.001. Error bars indicate standard deviation.

## Results

### Screening of drug library suggested CZ as a potential candidate drug to enhance the immune function of MSCs

**Fig. 1:**
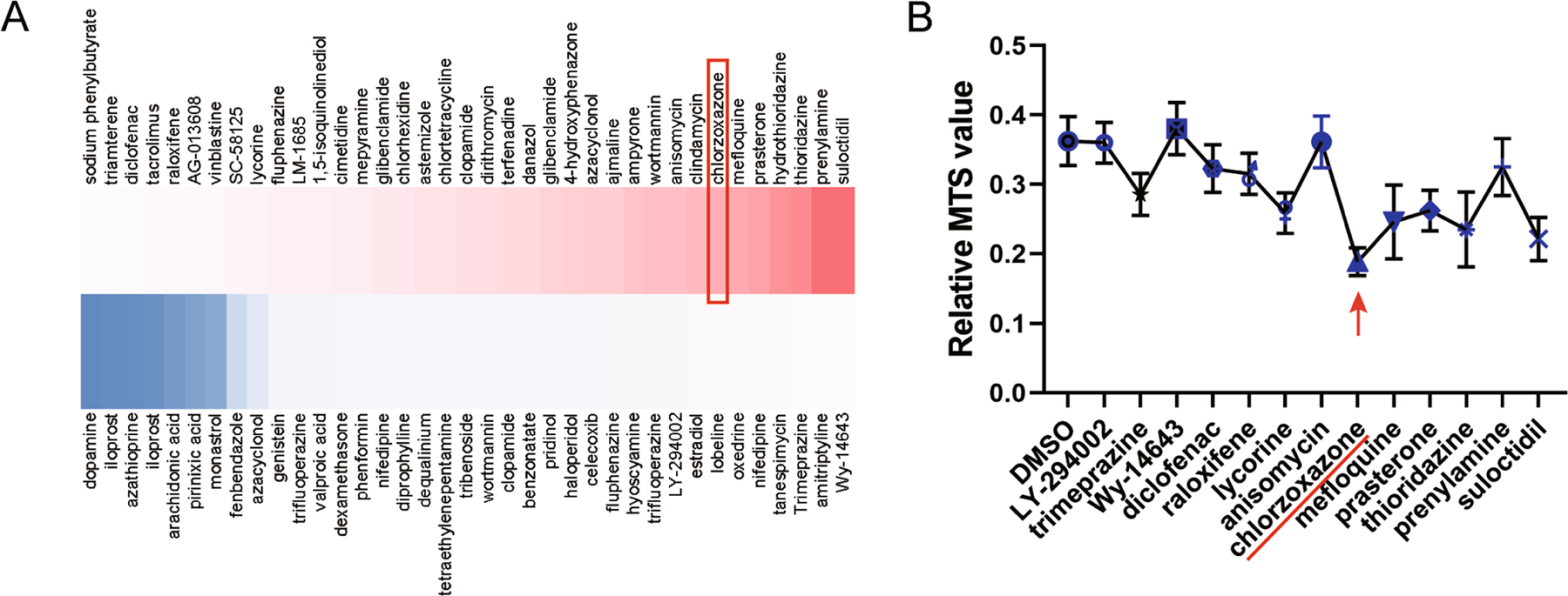
Candidate drugs screening from FDA-approved drug library. **a** Heatmap of screened drugs and gradation colored by folds of correlation (dark blue lowest and dark red highest). **b** Selective MTS proliferation assay of T cells co-cultured with MSCs pretreated with different drugs for 24 h.

We combined big data mining and biological function test when screening potential drugs from FDA-approved drug library. Computer graded the correlation between drugs and immune function and dozens of small molecular compounds were selected out and ranked by their score of relevance (Fig. 1a). The classical method to test MSCs immunosuppressive ability is co-culture of MSCs with peripheral blood mononuclear cells (PBMCs) and examine T cells activation and proliferation^30^. We confirmed the immunoregulatory function of these drugs by pretreatment to MSCs (derived from human umbilical cord, hereinafter inclusive) for 24 h followed by co-culture with PBMCs for 2 days in vitro. Among them, CZ appeared most outstanding indicated by the MTS proliferation assay (Fig. 1b).

### CZ changes no biological characteristics of MSCs

MSCs are characterized both morphologically and biologically by international consensus. Several criteria must be met to render cells eligible for cell therapy in clinical trial^31^. We observed that CZ-treated MSCs remained plastic adherent and resembled fibroblasts under optical microscope (Supplementary Fig. S1a), high expression CD73, CD90, CD105, and lack expression of CD34, CD45, and HLA-DR (Supplementary Fig. S1b) just like ordinary MSCs. These CZ pretreated MSCs could also differentiate into osteoblasts and adipocytes under specific osteogenic or adipogenic inductive culture conditions in vitro and exponentially express osteogenesis- and adipogenesis-associated genes, ALP, OPN, and RUNX2 and LPL, PPARγ, and CEBPα, respectively during the induction process (Supplementary Fig. S1c, d). Therefore, we confirmed that our CZ-primed MSCs still qualified the standard criteria to execute further study and ultimate medical application.

### MSCs maintain low immunogenicity after CZ treatment

**Fig. 2:**
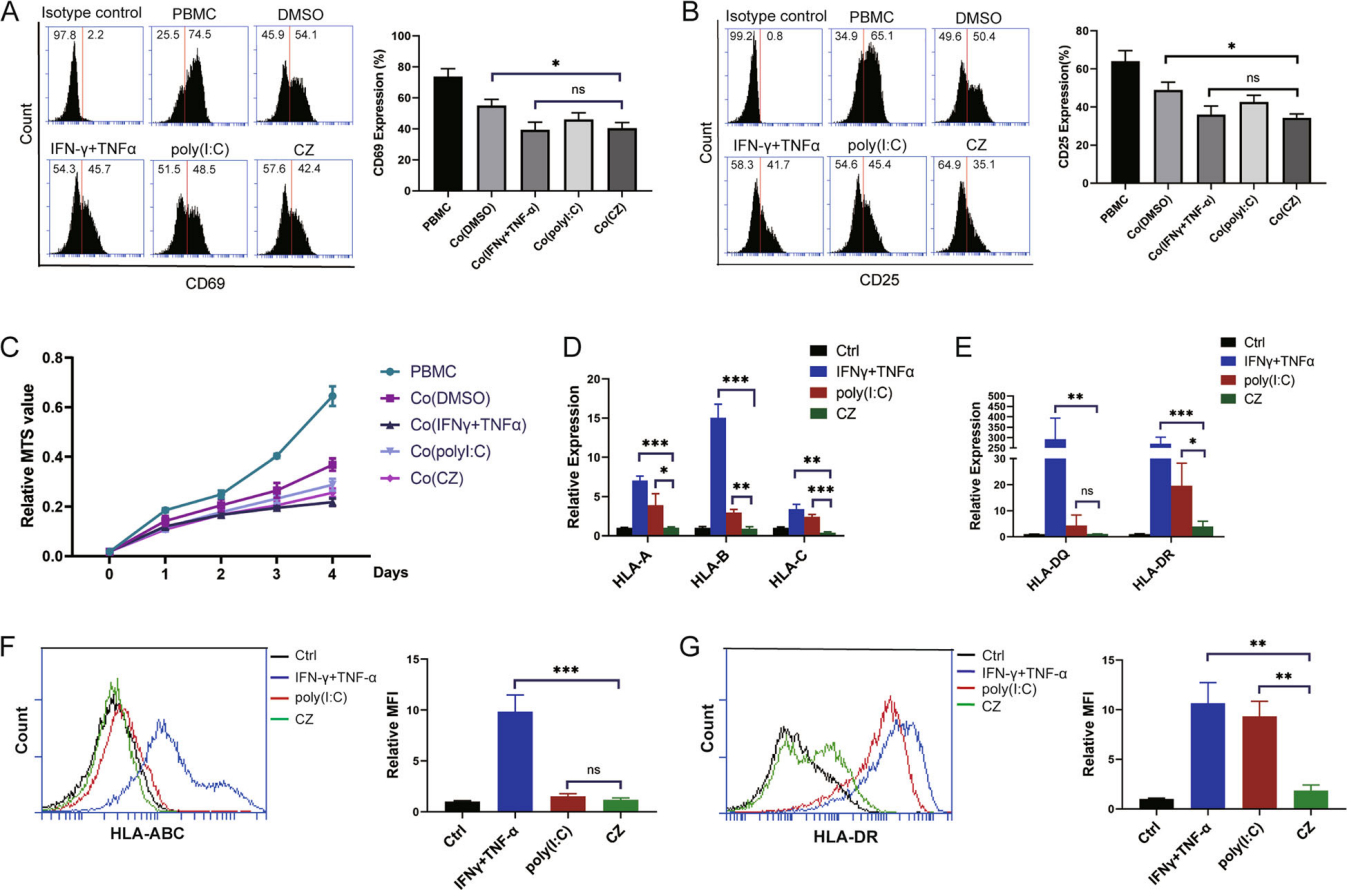
CZ-treated MSCs maintain low immunogenicity. **a**, **b** Representative flow cytometry analysis of T-cell activation marker CD69 (early stage)/CD25 (mid stage) activated by 50 μg/ml PHA for 24/48 h (left) and statistical histogram of CD69/CD25 expression on T cells (right) in different culture groups, respectively. **c** MTS assay of T cells proliferation in different culture groups for 4 days. **d** Histogram of gene expression of HLA-A, HLA-B, and HLA-C on MSCs treated by different regents for 24 h. **e** Histogram of gene expression of HLA-DQ and HLA-DR on MSCs treated by different regents for 24 h. **f**, **g** Representative flow cytometry test result of HLA-ABC/HLA-DR on MSCs treated by different regents for 24 h (left) and histogram of relative MFI (mean fluorescence intensity) of this flow cytometry test (right). *P < 0.05, **P < 0.01, ***P < 0.001, ns no significant difference.

To find out the advantages of CZ over other reagents, we compared it with IFN-γ and poly(I:C), two traditional molecules currently used to induce anti-inflammatory MSC2 and the induction protocol of MSC2 with these two reagents is quite established by international authority^22^. T cells activation and proliferation conducted by flow cytometry and the MTS assay as previously described clearly showed that CZ-treated MSCs possess similar immunosuppressive capacity with IFN-γ (20 ng/ml) primed MSCs and even better than those MSCs treated by poly(I:C) (1 μg/ml) (Fig. 2a–c). More importantly, by conducting quantitative RT-PCR and flow cytometry, we found that unlike IFN-γ which robustly induced expression of class I major histocompatibility complex (MHC I) and especially class II major histocompatibility complex (MHC II) on MSCs upon stimulation, CZ had nearly no effect on promoting HLA-A, HLA-B, HLA-C, and HLA-DQ, HLA-DR expression whereas poly(I:C) made relatively milder influence on these immunogenicity markers on human cells (Fig. 2d–g). Interestingly, the expression of vascular cell adhesion molecule (VCAM) was also elevated in IFN-γ-primed MSCs detected by flow cytometry analysis (Supplementary Fig. S2a). We thus concluded that MSCs maintain low immunogenicity upon CZ stimulation which can be critical in clinical treatment of immune disorders.

### CZ-treated MSCs strongly inhibit T cells activation and proliferation with distinct alterations in IDO and inflammatory cytokines

**Fig. 3:**
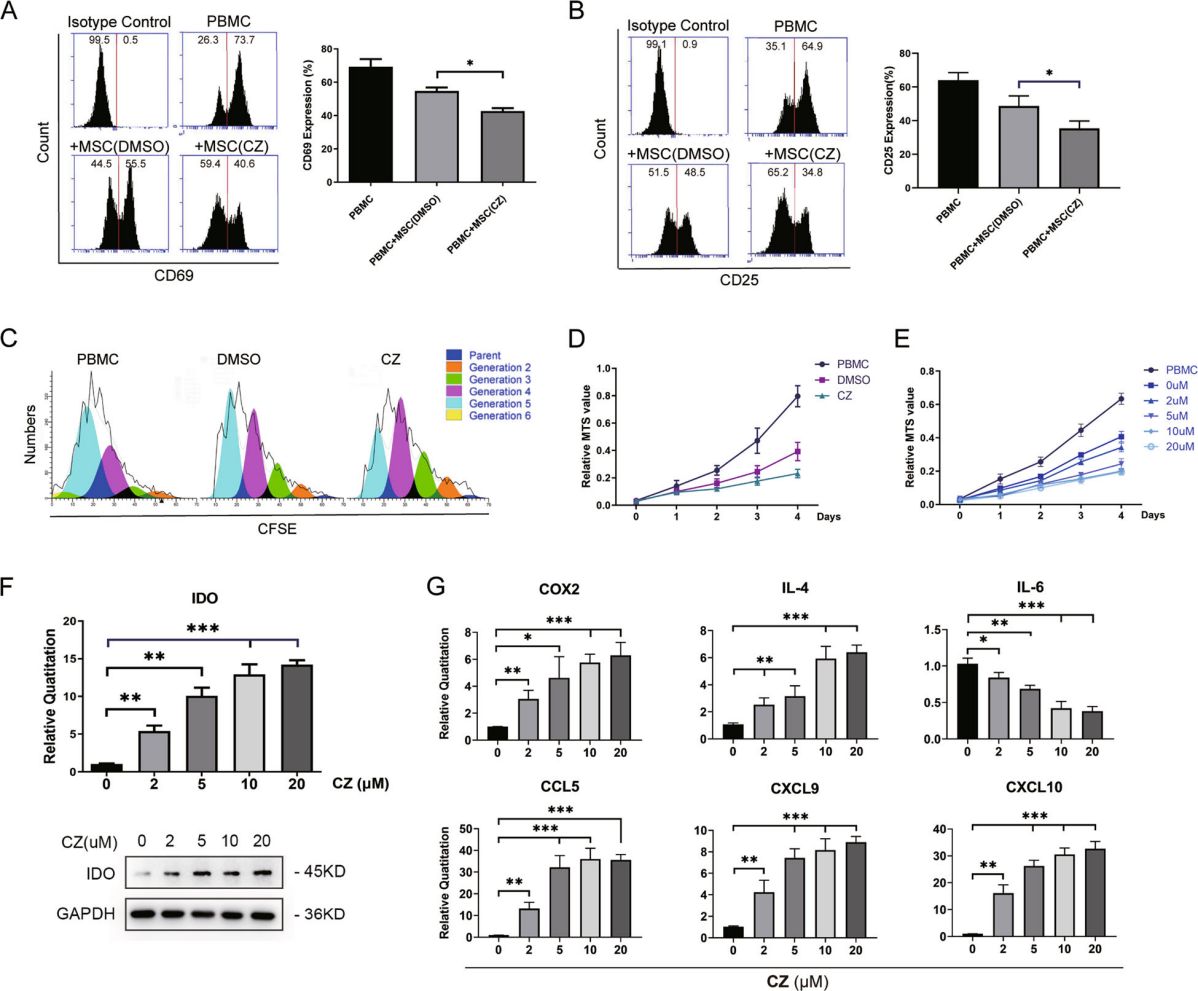
CZ enhances MSCs immunosuppressive function in vitro. **a**, **b** Representative flow cytometry analysis of T-cell activation marker CD69 and CD25 (left) and statistical histogram of CD69 and CD25 expression on T cells (right) in different culturing groups. **c** Histogram of generations of T cells in different culturing groups measured by the CFSE assay in 3 days. **d** MTS assay of T cells proliferation in different culture groups for 4 days. **e** MTS assay of T cells co-cultured with MSCs treated by different concentrations of CZ for 4 days. **f** Histogram of quantitative RT-PCR of RNA expression (up) and western blotting of protein expression (down) of IDO in MSCs treated by different CZ concentrations. **g** Histogram of quantitative RT-PCR of gene expression of main cytokines and chemokines altered by different CZ concentrations in MSCs. *P < 0.05, **P < 0.01, ***P < 0.001.

In order to investigate the effect of CZ on the immune function of MSCs, the inhibition of MSCs on PHA-induced T-cell activation was analyzed and indicated by CD69 (early-stage marker of T-cell activation) and CD25 (mid-stage marker of T-cell activation). Flow cytometry assay showed that CZ pretreated MSCs significantly suppressed the activation of T cells more effectively, compared with control group (DMSO-treated MSCs) (Fig. 3a, b). The proliferation rate of T cells dramatically decreased after co-culturing with CZ-treated MSCs, measured by fluorescent living cell staining dye CFSE for 3 days co-culturing with MSCs and the continuous MTS assay for 4 days, respectively (Fig. 3c, d). Furthermore, the effect seemed dose dependent as the suppressive ability of CZ-treated MSCs increased when applying incremental concentrations of the drug from 2 to 20 μM (Fig. 3e).

IDO is a key functional enzyme that catalyzes the rate-limiting step in tryptophan catabolism to N-formyl-kynurenine, thus contributing to MSCs immune suppression process in human and believed to be induced to express highly by IFN-γ which mostly secreted by lymphocytes and macrophages^32^. We conducted quantitative RT-PCR and western blotting to demonstrate that CZ could promote IDO expression both in transcriptional and translational levels, independent from additional IFN-γ (Fig. 3f). Other immune-related cytokines and chemokines like COX2, IL-4, HGF, TSG-6, CCL5 (RANTES), CXCL9, and CXCL10 (IP-10), synchronizing with IDO in MSC-mediated immune modulation, went through similar upregulation in RNA level, exclusive of IL-6 which was significantly downregulated during this process (Fig. 3g and Supplementary Fig. S3a). Clearly, the augmentation of MSCs immunomodulatory ability had positive correlation with concentration of CZ added in culture medium. Considering experimental efficiency, we chose the median concentration 10 μM as the optimal one in following experiments.

Since CZ can induce gene expression of Th2 secrotome in MSCs, we examined whether CZ can directly polarize Th cells into Th2 by introducing cytokine induction system to induce different subtypes of Th cells. It displayed that CZ can slightly polarize naïve Th cells into Th2 and obviously boosted the polarization effect when combined with cytokine induction system. Interestingly, for Treg induction, CZ only synergized with cytokine induction system and augmented the differentiation immensely (Supplementary Fig. S4a, b). However, CZ had nearly no influence on Th1 induction (data not shown). Although, CZ can polarize Th cells into Th2 and Treg to directly modulate immune system, it may also create a high-risk tumorigenesis niche in human body. On the contrary, CZ-primed MSCs give us a moderate choice for immune suppression since MCSs can adjust their immune status according to local immune environment. Therefore, we focused on MSCs to study further.

### CZ regulates phosphorylation of transcription factor FOXO3 independent of classical AKT or ERK signaling pathways

We have proved that CZ can enhance the immunosuppressive function of MSCs. Previous studies indicated that the mechanism may involve in transcription factor interferon regulatory factor 8 (IRF-8), FOXO3, and transmembrane signaling protein DNAX-activation protein 12 (DAP12)^33^. Our experiments showed there was no significant change in either RNA or protein levels of these three genes after CZ treatment in MSCs (Supplementary Fig. S4a, b).

**Fig. 4:**
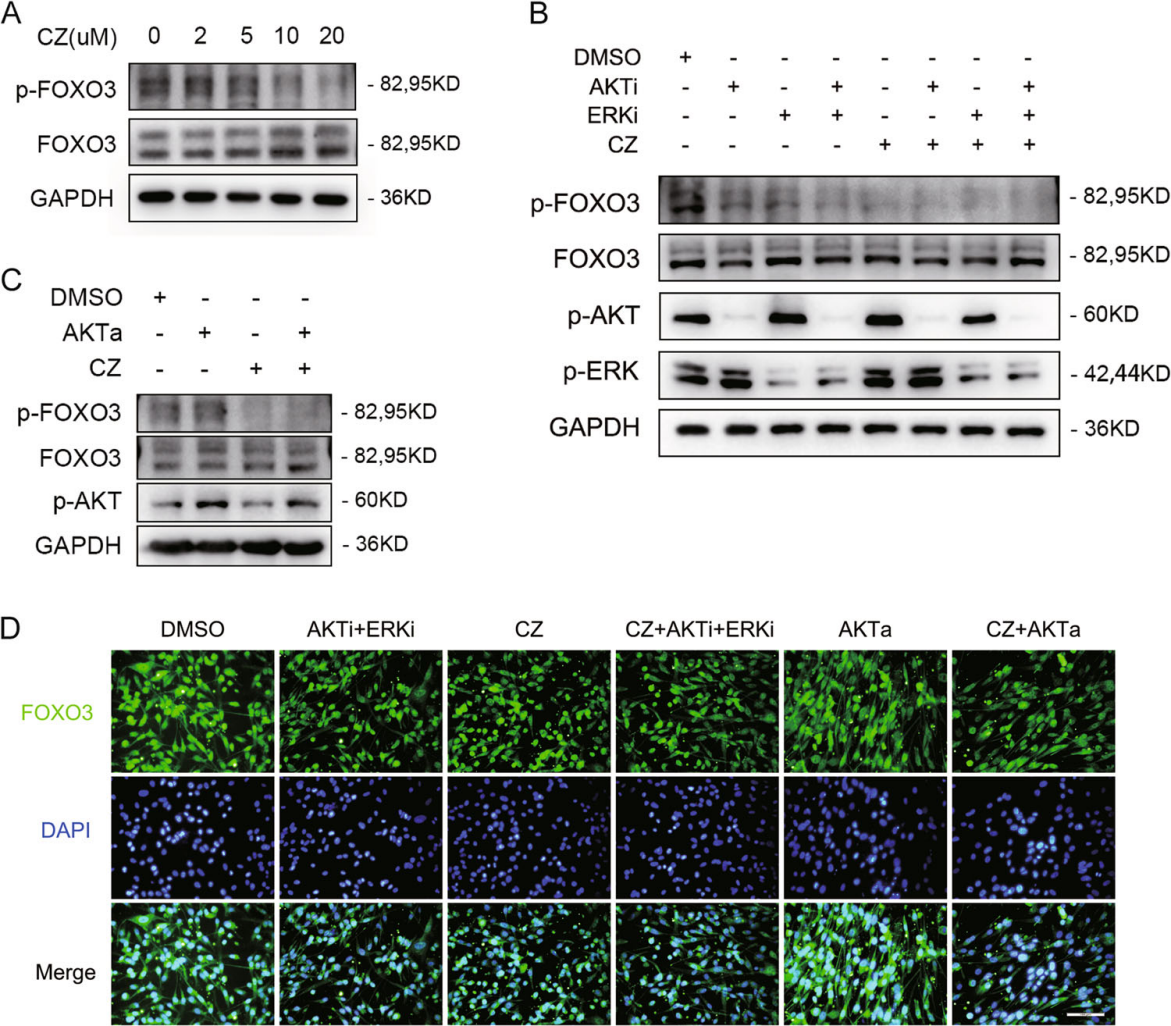
FOXO3 phosphorylation regulated by CZ. **a** Western blotting of p-FOXO3 and total FOXO3 influenced by CZ dose gradation. One gel was run and two blots were made in this image. One blot for total protein and the other for phosphorylated protein. The same loading control of GAPDH was used as Fig. 3f. **b** Western blotting of p-FOXO3, total FOXO3, p-AKT, and p-ERK influenced by CZ, AKT, and/or ERK inhibitors. Two gels were run and two blots were made in this image. One blot for total protein and the other for phosphorylated protein. **c** Western blotting of p-FOXO3, total FOXO3, and p-AKT influenced by CZ and/or AKT activators. One gel was run and two blots were made in this image. One blot for total protein and the other for phosphorylated protein. All protein bands were visualized by chemiluminescent ECL reagent without antibody stripping procedure. **d** Immunofluorescence of total FOXO3 cellular location influenced by CZ and/or different kinase inhibitors and activators. Scale bars: 100 μm.

It is also reported that MSCs execute immune functions relying on PI3K–AKT–FOXO3 and MAPK–ERK–FOXO3 signaling pathways^32,34^. To reveal the underlying mechanism, we examined staple kinases through these cascades and discovered there was a tight connection between CZ and FOXO3 transcription factor. Western blotting analysis showed that as concentration of CZ increased, total FOXO3 protein expressed higher accordingly whereas p-FOXO3 decreased (Fig. 4a). Considering phosphorylated FOXO3 protein translocated out of nucleus to cell plasma to be degraded, it is possible that the total FOXO3 protein increased because of less p-FOXO3 protein consumption in cytoplasm. We further studied CZ influence on AKT and ERK, the dominating two kinases to phosphorylate FOXO3^35,36^ but there was no significant change (data not shown). By adding AKT and ERK inhibitors (GSK2141795 for AKT and FR180204 for ERK), we found that FOXO3 is the ultimate target of CZ, independent of either AKT or ERK signaling pathways (Fig. 4b). AKT activator (SC 79) was then added to the culture system to further confirm CZ function and results. It displayed that CZ could rescue overphosphorylation of FOXO3 by highly activated AKT (Fig. 4c). Immunofluorescence demonstrated consistent outcome with western blotting that CZ inhibited FOXO3 protein phosphorylation like AKT and ERK inhibitors and rescued AKT activator caused overphosphorylation, thus retains FOXO3 inside nucleus to exert function of transcribing downstream genes (Fig. 4d). By adding AKT activator, we also found that CZ can slightly downregulates p-AKT (Fig. 4c), it is possible that CZ also works on AKT or upstream of AKT signaling.

### CZ inhibits phosphorylation of FOXO3 to enhance immunosuppressive capacity of MSCs

**Fig. 5:**
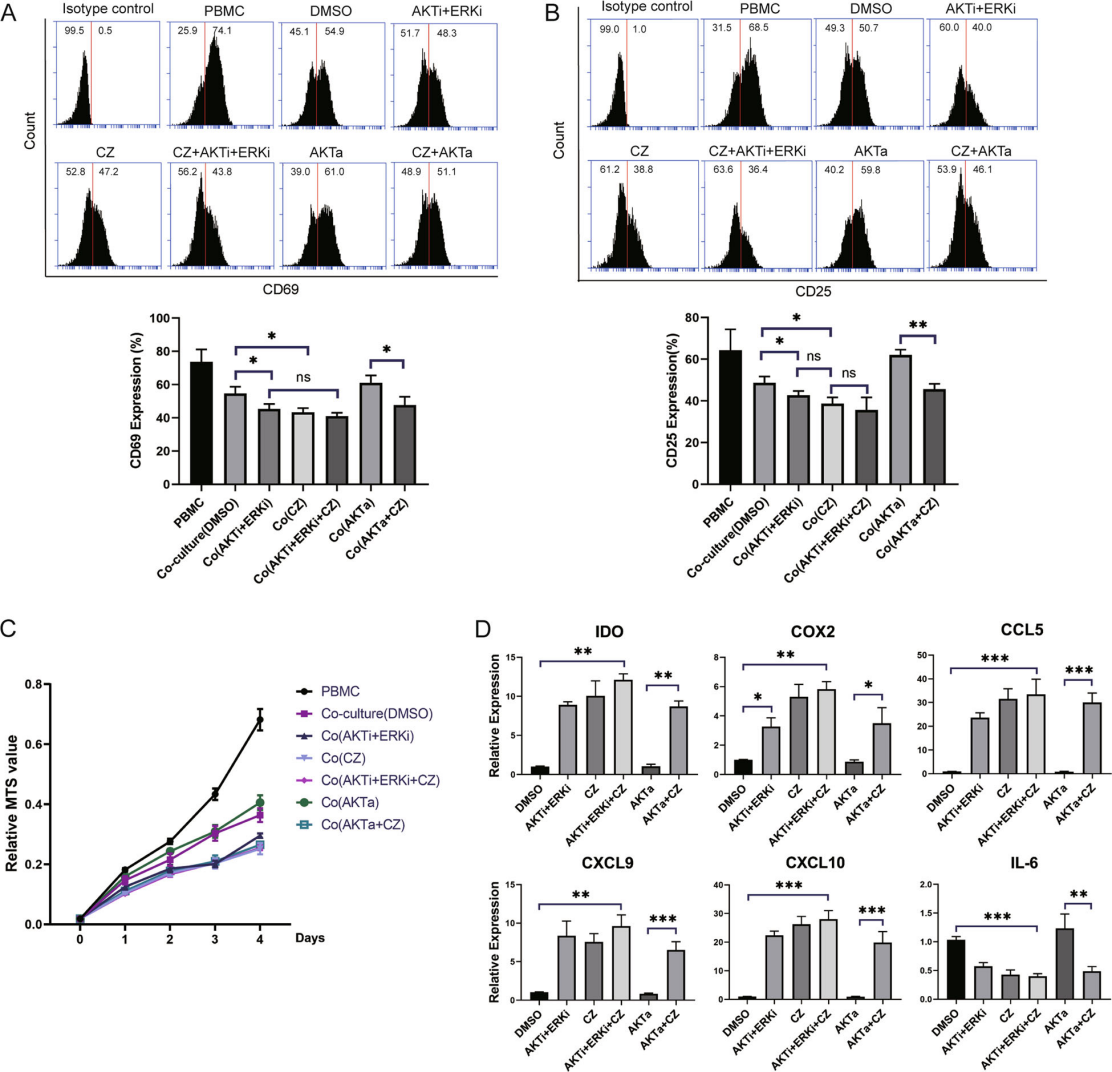
CZ enhances immunosuppressive ability of MSCs by inhibiting FOXO3 phosphorylation. **a**, **b** Representative flow cytometry analysis of T-cell activation marker CD69 and CD25 (up) and statistical histogram of CD69 and CD25 expression on T cells (down) in different culture groups. **c** MTS assay of T cells proliferation in different culture groups for 4 days. **d** Histogram of quantitative RT-PCR of gene expression of IDO and selected cytokines and chemokines secreted by MSCs altered by different CZ and/or different kinase inhibitors or activators. *P < 0.05, **P < 0.01, ***P < 0.001, ns no significant difference.

To further confirm the target site of CZ, examine the role FOXO3 played in immune suppression and compare biological functions between AKT/ERK inhibitors and CZ, functional studies in vitro were conducted as follows. T cells activation and proliferation measured by flow cytometry and the continuous 4-day MTS assay respectively showed clearly that CZ-treated MSCs suppress equal percentage of T cells with AKT and ERK inhibitors, if not higher. And CZ could reverse dysfunction of MSCs suppression on T-cell activation caused by AKT activator (Fig. 5a–c). Expression of immune mediators measured by quantitative RT-PCR displayed similar pattern that CZ induces steady and potent IDO, cytokines, and chemokines expression of MSCs in contrast with those kinase inhibitors or activators (Fig. 5d and Supplementary Fig. S3d). Therefore, we demonstrated that in comparison with AKT and ERK inhibitors, CZ exhibited relatively stronger effect in promoting immunosuppressive capacity of MSCs by inhibiting FOXO3 phosphorylation.

### CZ promotes immune suppression ability of MSC in vivo

**Fig. 6:**
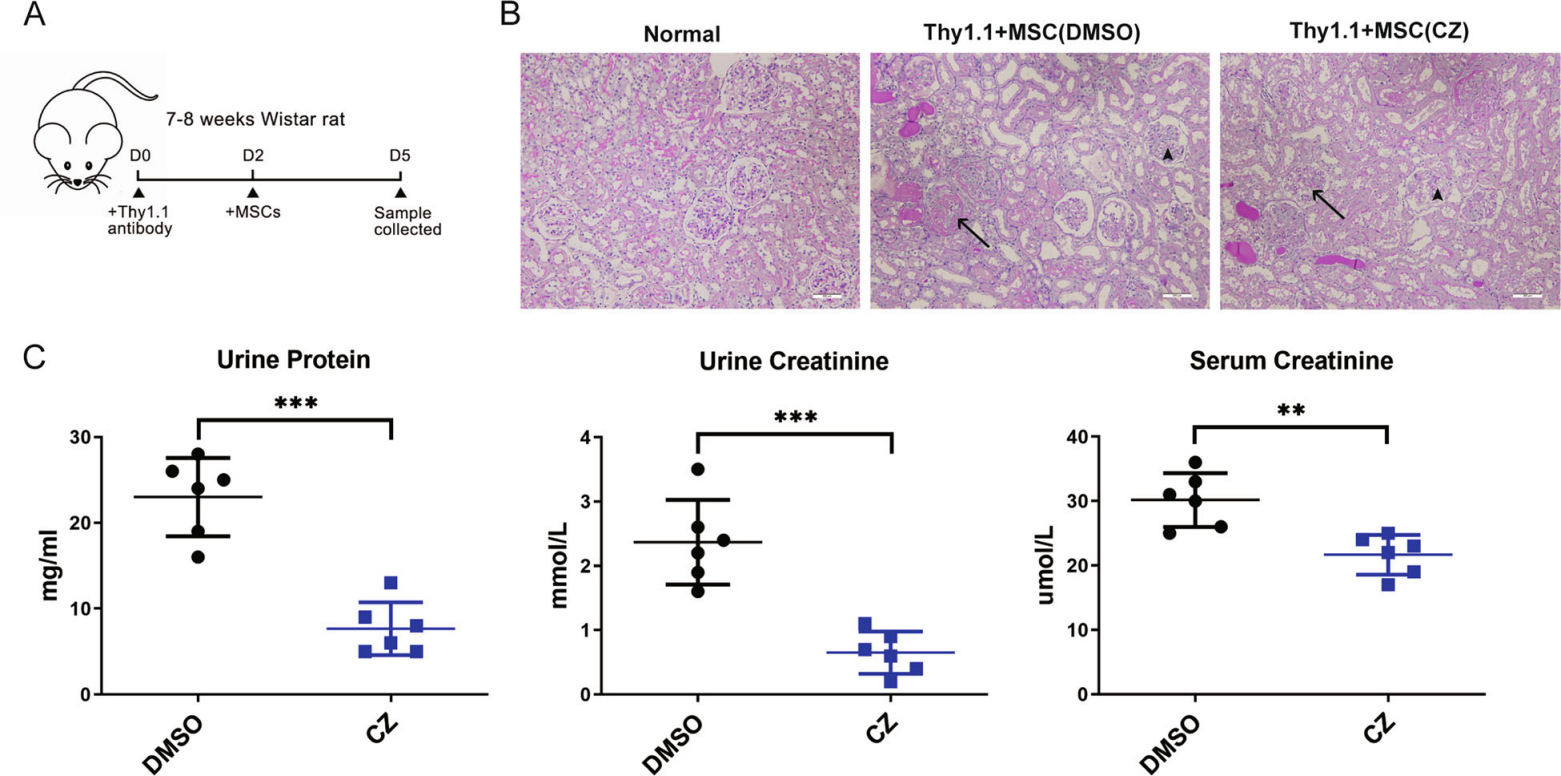
CZ promotes immunosuppressive ability of MSCs in rat model. **a** Schematic diagram of acute kidney injury rat model. **b** PAS staining of pathological section of kidney tissue of normal rat and rat model administered by DMSO or CZ-treated MSCs, respectively. Arrows indicate fibrinoid necrosis (long arrows) and mesangial cell proliferation (short arrows) of glomeruli, respectively. **c** Individual maps of urine protein, urine creatinine, and serum creatinine in acute kidney injury rat model administered by DMSO or CZ-treated MSCs, respectively. *n* = 6, **P < 0.01, ***P < 0.001, scale bars: 200 μm.

Thy1.1 induced acute kidney injury is a well-established model of acute immune response causing renal cells apoptosis^37^. To confirm the function of CZ in promoting immunosuppressive capacity of MSCs in vivo, we employed an acute kidney injury rat model induced by Thy1.1 antibody. Two days after Thy1.1 antibody injection, MSCs were infused intravenously and blood and urine samples were collected after another 3 days prior to kidney excision (Fig. 6a). Pathological section of kidney stained by PAS staining showed that CZ-treated MSCs could alleviate inflammatory infiltration in renal tissue and lessen fibrinoid necrosis of glomeruli more effectively (Fig. 6b). Biochemical tests of blood and urine of model rats showed unanimous result that urine protein, serum creatine, and urine creatine, three key indicators to exhibit inflammation degree of kidney, were all significantly decreased compared with DMSO-treated control group. (Fig. 6c). Therefore, we concluded that CZ promoted immunosuppressive ability of MSCs to better cure acute inflammatory response in rat.

## Discussion

MSCs hold potent capacity of immunomodulation to treat immune-related disorders, making it very promising for cell therapy. However, MSCs’ immunoregulatory function can be unsteady and equivocal for patients under different immune conditions. Apart from individual difference, it is highly possible that primordial MSCs derived from healthy donors can change their immune properties to adapt recipients’ body condition, polarizing into proinflammatory MSC1 or anti-inflammatory MSC2 phenotype^23^. Some patients’ immune status is not optimal for MSCs to perform their required abilities. Therefore, it is strongly necessary to stimulate primitive MSCs into an applicable state or phenotype, in most cases MSC2, prior to administer to recipients. In this study, we successfully screened out a small molecule drug CZ which can induce MSCs into anti-inflammatory MSC2 in vitro and promote immunosuppression ability of MSCs in vivo.

Previous studies have shown that, MSC2 can only be induced by IFN-γ and poly(I:C)^34^. IFN-γ has long been proved to be a robust stimulator to activate MSC to exert their immunosuppressive function as MSC2. Together with TNF-α, IL1α, or IL1β, IFN-γ boosts immune suppression capacity of MSCs intensely by promoting MSCs to express large amount of immune mediators such as IDO (or NO in mice) and PGE2^23^. The advantages of IFN-γ are notable but the defects are also intrusive. IFN-γ-primed MSCs highly express class I and class II MHC molecules i.e., HLA-A, HLA-B, HLA-C, HLA-DP, HLA-DM, HLA-DO, HLA-DQ, and HLA-DR in human body^24,30^, causing much higher immunogenicity of MSCs which can be a non-negligible obstacle for medical treatment. Poly(I:C) is an agonist for toll-like receptor 3 and can induce MSCs into anti-inflammation type MSC2 likewise, only less intensive than IFN-γ^21^. However, poly(I:C) is a synthetic double-stranded RNA which mimics structure and pathological function of virus, rendering it toxic and dangerous for human body. In contrast to IFN-γ and poly(I:C), we demonstrated that CZ did not elevate MHC I and MHC II expression, keeping MSCs in relatively low immunogenicity and did not have severe pathological effects on human body as a drug approved by government authorities.

**Fig. 7:**
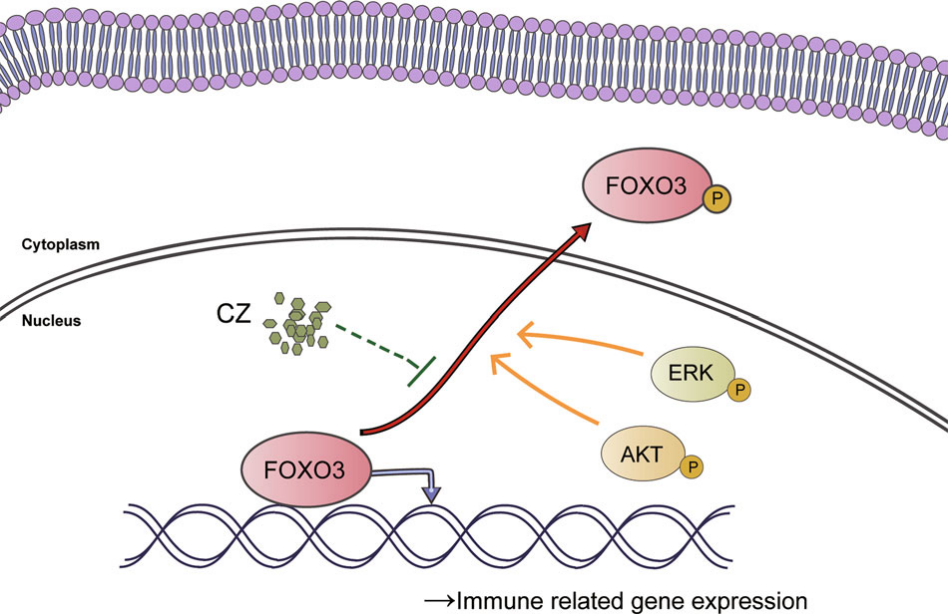
Schematic diagram of CZ-mediated FOXO3 signaling. CZ inhibits FOXO3 phosphorylation thus prevent FOXO3 from translocating to cytoplasm, retaining FOXO3 inside nucleus to transcript downstream genes.

As an FDA-approved drug, CZ is currently used for muscle relaxation, mainly acting on the spinal cord by depressing reflexes^38^. It also exists as a marker of hepatic cytochrome P4502E1 in humans^39^. In our study, we successfully proved CZ is nearly as effective as IFN-γ for MSC priming to induce anti-inflammatory MSC2, meanwhile keeping basic biological characteristics and functions of MSCs. Our discovery has demonstrated a novel usage of CZ and revealed the mechanism of CZ in the immunomodulatory process, entitling this classical drug a brand-new value in healthcare. As an authorized safe drug, we believe CZ is right ready for bedside use to benefit patients (Fig. 7).

In order to better explore the immunoregulatory function of CZ, we further investigated potential signaling pathway involved in it. Researchers have discovered that IDO and other immune mediators are promoted by FOXO3 and IRF-8 but suppressed by DAP12^33^. Among them, FOXO3 was previously proved to be related to muscle atrophy^40^, which may connect to the original function of CZ as muscle relaxant. These evidences gave us clues to dig and explain the intrinsic mechanism of CZ in stimulating MSCs into MSC2. Thus, we examined these transcription factors and found that FOXO3 phosphorylation was inhibited in MSCs after treatment by CZ as expected, suggesting that FOXO3 might be involved in the CZ-mediated regulation of the immune function of MSCs.

FOXO3 is a transcription factor belonging to the O subclass of the forkhead family. It is thought to be associated with tumorigenesis, immunoregulation, and even longevity of humans^41–45^. Besides, researchers discovered that FOXO3 regulates neurogenesis in adult by interacting with ten-eleven translocation-2 protein^46^ and maintains redox balance in neural stem cells coordinating metabolic pathways^47^. Others found that FOXO3 activates autophagy during osteogenic differentiation^48^ and protects hematopoietic stem cells from metabolic stress by autophagy^49^. Importantly, FOXO3 is reported to be phosphorylated mainly by p-AKT along PI3K–AKT signaling and p-ERK through MAPK–ERK signaling in previous studies on cell survival and acute myeloid leukemia, respectively^35,36^. Our experiments indicated that CZ inhibits FOXO3 phosphorylation independent of classical PI3K–AKT (or only slightly rely on AKT signaling) and MAPK–ERK signaling pathways, and we demonstrated that CZ promotes expression of immunomodulatory factors via inhibition of p-FOXO3. However, the exact pathway from membrane receptor to the specific downstream target genes of MSCs remains unclear, future study may focus on this question to further understand the biological function of CZ.

We proved that CZ can directly enhance the polarization of Th cells into Th2 and Treg cells and the influence on Treg is significant. Since a fulminant Th2 and Treg niche may contribute to cancer initiation or formation^50,51^, a systemic treatment of CZ for patient is not recommended. However, CZ can be a quite excellent stimulator in vitro to enhance immune regulating function of MSCs. We understand that MSCs can adjust their immune status responding to the microenvironment and keep an immune balance of hosts. It is unlikely that CZ-primed MSCs create a tumor-initiating niche as an over immune suppressed environment may polarize MSCs into proinflammatory MSC1^23^. Therefore, we can simply add CZ in culture medium of MSCs prior to body infusion by repeatedly washing MSCs after treatment. Some minimum of CZ residue may exist after washing but the safety is certainly more guaranteed than IFN-γ and Poly(I:C).

Our study gives solid evidences of CZ’s role on enhancing immunoregulatory function of MSCs in vitro and in vivo, establishing a novel, effective, and simple approach to induce immunosuppressive MSC2. Further research should be conducted to examine effects of CZ as well as roles of FOXO3 played in other animal models and in larger scale, hopefully paving way for clinical trials in the future.

## Supplementary information

supplementary table 1

supplementary figure legends

supplementary figure S1

supplementary figure S2

supplementary figure S3

supplementary figure S4

supplementary figure S5
