# Supplementary material for: Chlorzoxazone, a small molecule drug, augments immunosuppressive capacity of mesenchymal stem cells via modulation of FOXO3 phosphorylation
Source: Cell Death Dis. 2020 Mar 2;11(3):158. doi: 10.1038/s41419-020-2357-8 (PMC7052156; doi:10.1038/s41419-020-2357-8)
Supplement: Supplementary file 1 — supplementary table 1 [file 41419_2020_2357_MOESM1_ESM.docx]

**Supplementary Table 1. Sequence of primers used in this study**

| **Gene** | **Primer sequence** (5’-3’) |
| --- | --- |
| **HLA-A** | F: AAAAGGAGGGAGTTACACTCAGG |
|  | R: GCTGTGAGGGACACATCAGAG |
| **HLA-B** | F: CAGTTCGTGAGGTTCGACAG |
|  | R: CAGCCGTACATGCTCTGGA |
| **HLA-C** | F: CCATGAGGTATTTGTGGACCG |
|  | R: TCTCGGACTCTCGTCGTC |
| **HLA-DQ** | F: TCGCTCTGACCACCGTGAT |
|  | R: AGGGACCGTAAAACTGGTACAA |
| **HLA-DR** | F: TCTGGCGGCTTGAAGAATTTG |
|  | R: GGTGATCGGAGTATAGTTGGAGC |
| **IDO** | F: TGCTTGGAGAAAGCCCTTCA |
|  | R: CGTCTGATAGCTGGGGGTTG |
| **COX2** | F: CCCTTCTGCCTGACACCTTT |
|  | R: TTCTGTACTGCGGGTGGAAC |
| **IL-4** | F: CGGCAACTTTGTCCACGGA |
|  | R: TCTGTTACGGTCAACTCGGTG |
| **IL-6** | F: ACTCACCTCTTCAGAACGAATTG |
|  | R: CCATCTTTGGAAGGTTCAGGTTG |
| **CCL5** | F: CAGTCGTCTTTGTCACCCGA |
|  | R: CGGGTGGGGTAGGATAGTGA |
| **CXCL9** | F: CCAGTAGTGAGAAAGGGTCGC |
|  | R: AGGGCTTGGGGCAAATTGTT |
| **CXCL10** | F: GTGGCATTCAAGGAGTACCTC |
|  | R: TGATGGCCTTCGATTCTGGATT |
| **ALP** | F: CCACGTCTTCACATTTGGTG  AGACTGCGCCTGGTAGTTGT |
|  | R: AGACTGCGCCTGGTAGTTGT |
| **OPN** | F: ACTCGAACGACTCTGATGATGT |
|  | R: GTCAGGTCTGCGAAACTTCTTA |
| **RUNX2** | F: TGTCATGGCGGGTAACGAT |
|  | R: AAGACGGTTATGGTCAAGGTGAA |
| **LPL** | F: TCATTCCCGGAGTAGCAGAGT  TCATTCCCGGAGTAGCAGAGT  TCATTCCCGGAGTAGCAGAGT  TCATTCCCGGAGTAGCAGAGT |
|  | R: GGCCACAAGTTTTGGCACC |
| **PPARγ** | F: CCTATTGACCCAGAAAGCGATT |
|  | R: CATTACGGAGAGATCCACGGA |
| **CEBPα** | F: AGGAACACGAAGCACGATCAG |
|  | R: CGCACATTCACATTGCACAA |
| **IRF-8** | F: ACGCTGTGCTTTGAATAAGAGC  TCCTCAGGAACAATTCGGTAAAC  ACTGAGACCGAGTCGCCTTAT  ATACGGCCTCTGTGTGTTGAG |
|  | R: TCCTCAGGAACAATTCGGTAAAC |
| **DAP12** | F: ACTGAGACCGAGTCGCCTTAT |
|  | R: ATACGGCCTCTGTGTGTTGAG |
| **FOXO3** | F: CGGACAAACGGCTCACTCT |
|  | R: GGACCCGCATGAATCGACTAT |
| **GAPDH** | F: GGTCACCAGGGCTGCTTTTA |
|  | R: GGATCTCGCTCCTGGAAGATG |
