# Supplementary material for: Chlorzoxazone, a small molecule drug, augments immunosuppressive capacity of mesenchymal stem cells via modulation of FOXO3 phosphorylation
Source: Cell Death Dis. 2020 Mar 2;11(3):158. doi: 10.1038/s41419-020-2357-8 (PMC7052156; doi:10.1038/s41419-020-2357-8)
Supplement: Supplementary file 2 — supplementary figure legends [file 41419_2020_2357_MOESM2_ESM.docx]

**Supplementary Figure Legends**

**Fig. S1 Biological characteristics of CZ-treated MSCs**

**a** Morphology of MSCs under optical microscope which resembles fibroblasts. **b** Flow cytometry test results of phenotype of MSCs. **c** Oil red O staining (left) at day12 and ALP staining (right) at day5 to indicate adipogenesis and osteogenesis of MSCs respectively. **d** Histogram of RNA Expression by Log10 transformation of adipogenesis related genes LPL, PPARγ and CEBPα and osteogenesis related genes ALP, OPN and RUNX2 in successive 9-day induction respectively. Scale bars: 200 μm.

**Fig. S2 IFN-γ promotes VCAM expression on MSCs**

Representative flow cytometry test result of adhesion molecule VCAM on MSCs treated by different regents for 24 h and histogram of relative MFI of this flow cytometry test (right). ** P＜0.01, ns: no significant difference.

**Fig. S3 Change of cytokines under different conditions**

**a** Histogram of RNA expression of HGF and TSG-6, two of the key indicators in MSC-mediated immune regulation, altered by different concentrations of CZ. **b** Histogram of RNA expression of IL-4, HGF and TSG-6 influenced by CZ and/or kinase inhibitors and activator. * P＜0.05, ** P＜0.01.

**Fig. S4 CZ polarize Th cells into Th2 and Treg**

**a** Flow cytometry of Th2 cells induced by CZ, cytokine system or CZ plus cytokine system. **b** Flow cytometry of Treg cells induced by CZ, cytokine system or CZ plus cytokine system.

**Fig. S5 RNA and protein expression of IRF-8, DAP12 and FOXO3**

**a** Histogram of RNA expression of IRF-8, DAP12 and FOXO3 with or without CZ treatment. **b** Western blotting of protein expression of IRF-8, DAP12 and FOXO3 with or without CZ treatment.
