# Supplementary figures and images for: Chlorzoxazone, a small molecule drug, augments immunosuppressive capacity of mesenchymal stem cells via modulation of FOXO3 phosphorylation
Source: Cell Death Dis. 2020 Mar 2;11(3):158. doi: 10.1038/s41419-020-2357-8 (PMC7052156; doi:10.1038/s41419-020-2357-8)

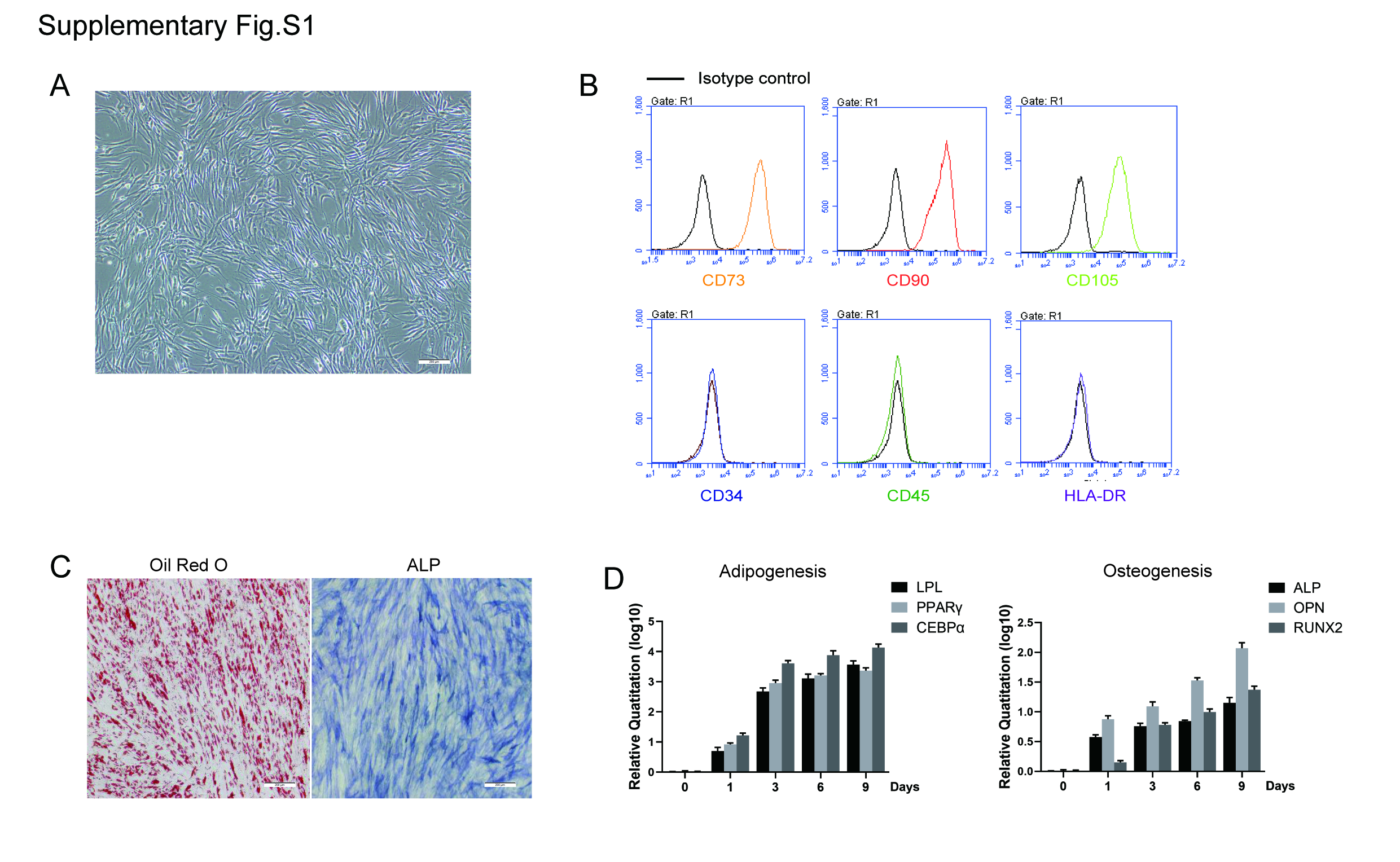

Supplement: Supplementary file 3 — supplementary figure S1 [file 41419_2020_2357_MOESM3_ESM.tif]

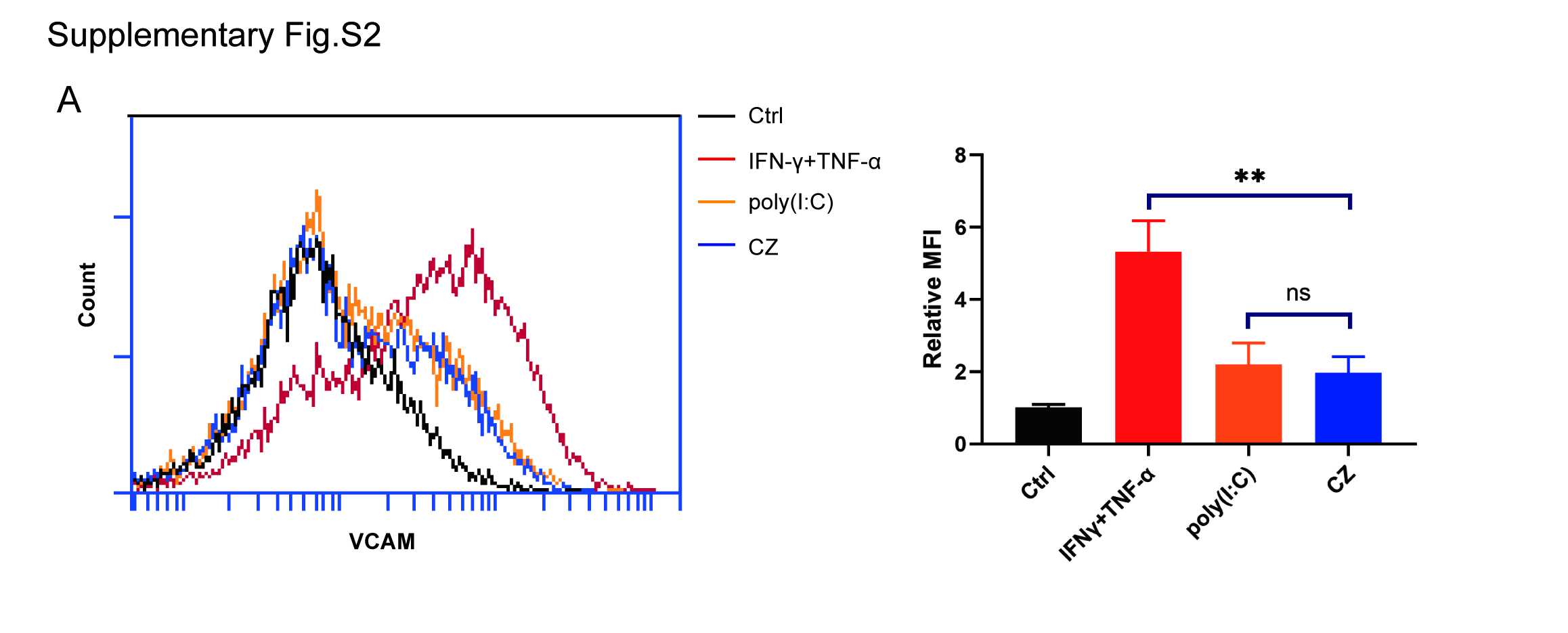

Supplement: Supplementary file 4 — supplementary figure S2 [file 41419_2020_2357_MOESM4_ESM.tif]

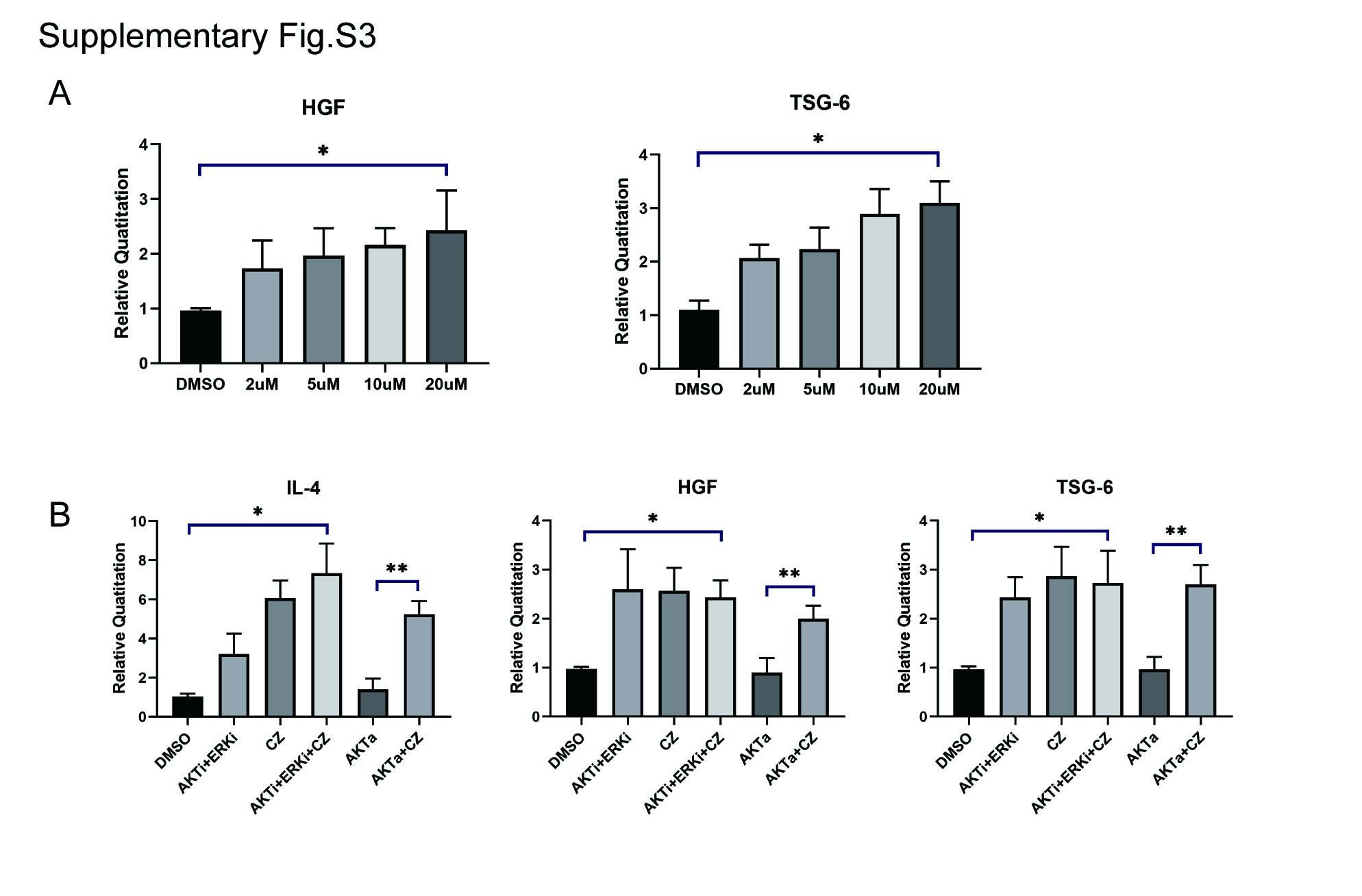

Supplement: Supplementary file 5 — supplementary figure S3 [file 41419_2020_2357_MOESM5_ESM.tif]

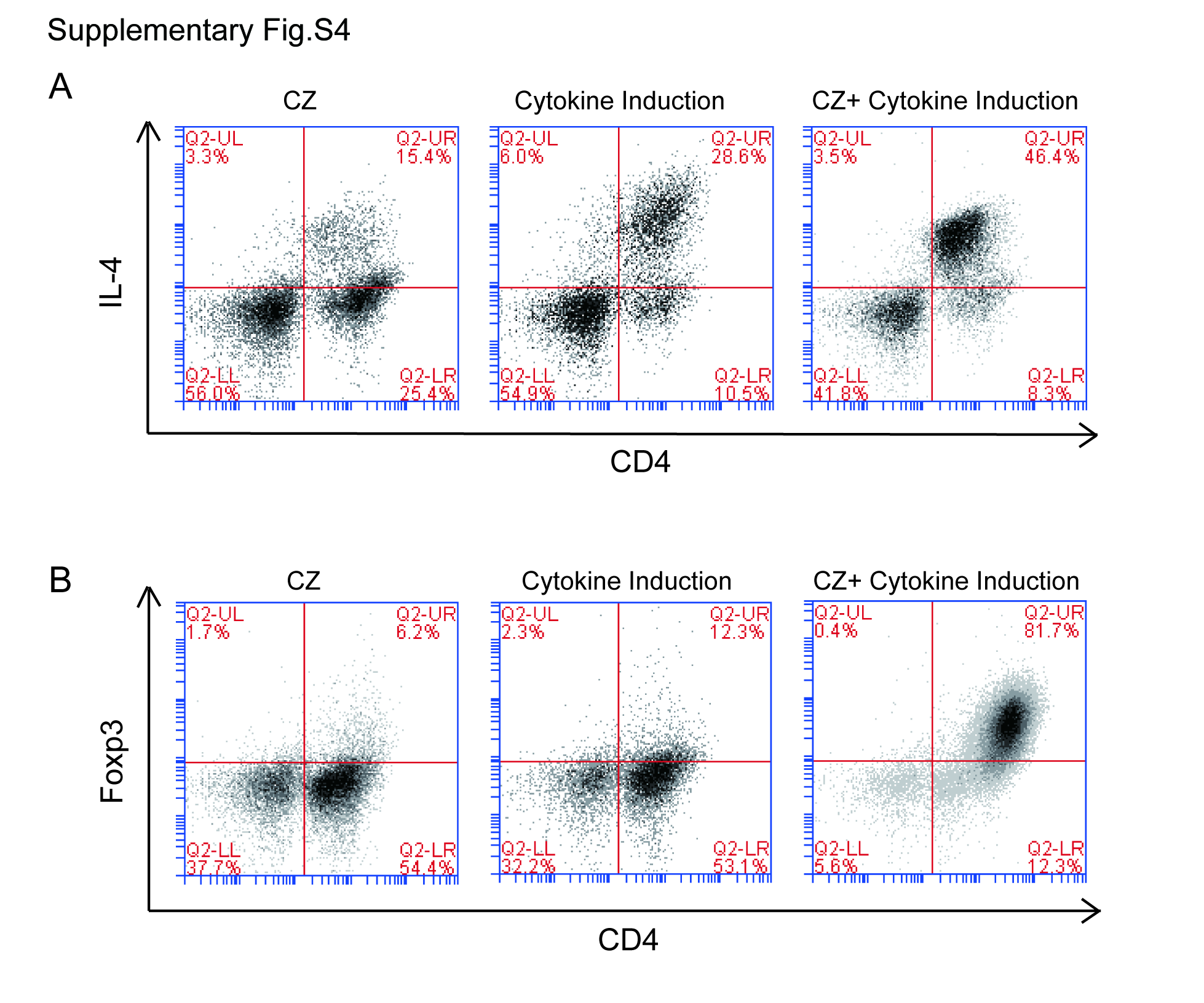

Supplement: Supplementary file 6 — supplementary figure S4 [file 41419_2020_2357_MOESM6_ESM.tif]

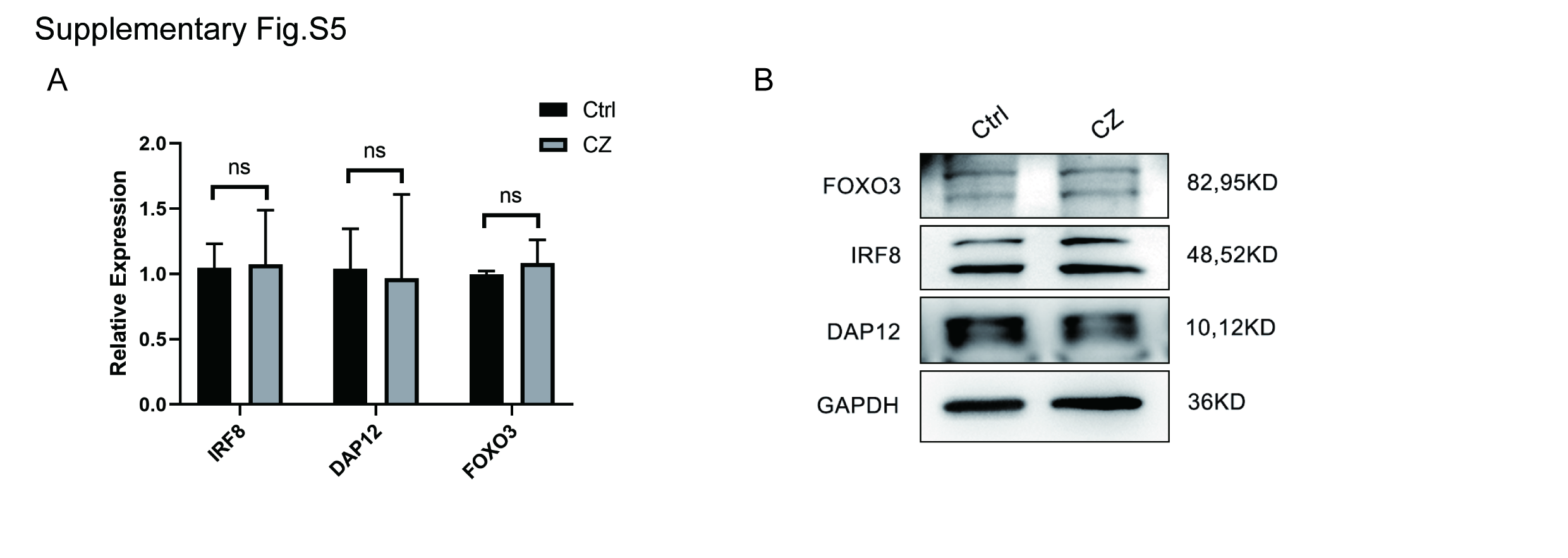

Supplement: Supplementary file 7 — supplementary figure S5 [file 41419_2020_2357_MOESM7_ESM.tif]
